# High-resolution modeling of extreme heat events with socioeconomic consideration: a real-case WRF–LES approach

**DOI:** 10.1007/s11356-025-36928-w

**Published:** 2025-09-12

**Authors:** Maryam Golbazi, Frank Liu, Yin-Hsuen Chen, Timothy W. Juliano, Heather Richter

**Affiliations:** 1 Old Dominion University and Thomas Jefferson National Accelerator Facility joint institute on Advanced Computing for Environmental Studies, Old Dominion University, 1070 University Blvd, Portsmouth, VA USA; 2 School of Data Science, Old Dominion University, 5115 Hampton Blvd, Norfolk, VA 23509 USA; 3 Center for Geospatial Science, Education, and Analytics, Old Dominion University, 4111 Monarch Way, Norfolk, VA 23508 USA; 4 U.S. National Science Foundation National Center for Atmospheric Research, Research Applications Laboratory, 3450 Mitchell Ln, Boulder, CO 80301 USA; 5 Institute for Coastal Adaptation and Resilience (ICAR), Old Dominion University, 800 West 46th Street, Norfolk, VA 23508 USA; 6 AiDASH, Palo Alto, CA USA

**Keywords:** Large-eddy simulation, Multiscale modeling, Nested mesoscale to large-eddy simulations, Urban Heat Island

## Abstract

The overarching goals of this work is to explore best practices for micro-scale modeling of a real case, identify relevant phenomena by using high-resolution modeling, and to explore their implications for public health, and climate resilience strategies in Hampton Roads, VA, USA. This project employs the Weather Research and Forecasting (WRF) model to conduct a comprehensive study of Hampton Roads, utilizing a coupled mesoscale to microscale modeling capable of resolving boundary layer turbulence. This study has three primary objectives: (1) to establish the optimal mesoscale to Large-Eddy Simulation (LES) configurations for complex geographical regions such as the Hampton Roads (HR) domain and address challenges inherent to multi-scale modeling; (2) as a demonstration, to identify extreme heat episodes and urban heat islands within the study area; and (3) to explore the correlation between these heat islands and the socio-economic characteristics of HR neighborhoods. Model performance was evaluated using observational data, applying standard statistical metrics such as correlation coefficient, mean bias, and root mean square error to select the most realistic model configuration. Similar statistical methods were used to assess the relationship between heat exposure and socio-economic factors. We also introduce a new metric, cooling energy demand, to quantify the potential economic burden of extreme heat. The Results show that lower-income communities are disproportionately exposed to higher heat levels and face greater cooling energy demands compared to rural areas. In addition, through extensive testing, we identified the cell-perturbation method as an effective approach for producing physically realistic LES simulations validated against observations. Future work will extend this approach to neighborhood-scale air quality modeling to develop a more comprehensive understanding of environmental stressors and support targeted climate resilience strategies for vulnerable communities.

**Supplementary Information:**

The online version contains supplementary material available at 10.1007/s11356-025-36928-w.

## Introduction and literature review

**Fig. 1:**
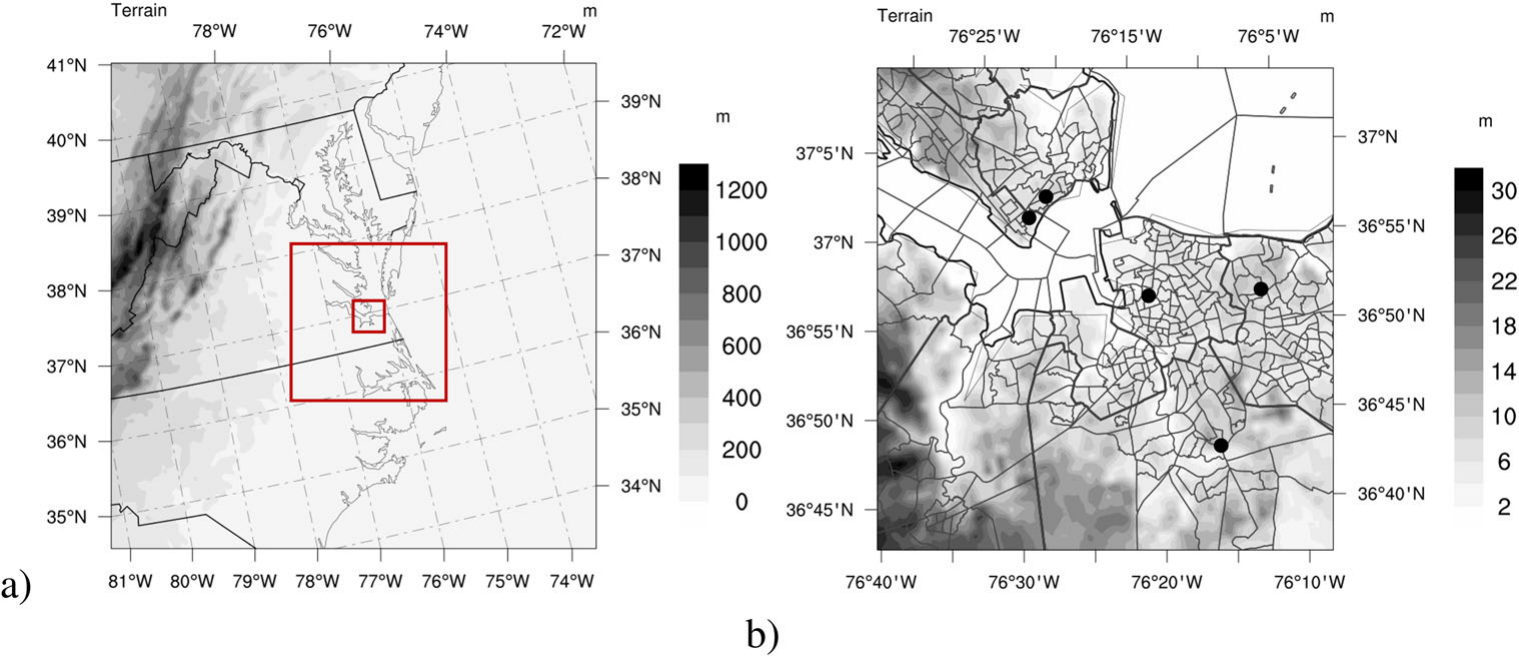
The study domain: **a** the outmost domain configured at 4050 m grid spacing, with nested domains shown with red boxes at 1350 m, and 150 m grid spacing, respectively, and **b** the innermost LES domain at 150 m grid spacing indicating the counties (with thicker black lines), and U.S. Census tracts (with thinner black lines). The black circles in **b** are the location of the PurpleAir sensors used for model performance analysis in the “Model performance evaluation” section

The Urban Heat Island (UHI) effect (Landsberg 1981; Oke 1982; Voogt and Oke 2003) refers to the temperature disparity between urbanized areas and their surrounding rural or natural environments. This phenomenon is primarily attributed to the dominance of artificial surfaces, such as pavements and buildings, which absorb and retain heat more efficiently than natural landscapes. The intensity of UHI can be evaluated using two closely related factors, namely, urban skin temperature, which measures the heat emitted by surfaces such as roads and roofs, and meteorological factors, which refer to the difference in air temperature between urban and rural areas (Oke 1982). In densely built environments, particularly those with limited green spaces and high-rise buildings, short-term temperature deviations exceeding 12 degrees Celsius have been documented. For example, Tokyo has experienced such extreme variations, demonstrating the significant impact of urban infrastructure on local temperatures (Tran et al. 2006). The UHI effect is observed in most major global cities, with its intensity varying depending on factors such as population density, urban layout, and local climate conditions (Phelan et al. 2015).

Anthropogenic heat refers to heat generated by human activities, including electricity, fuel consumption, and metabolic heat production. Anthropogenic materials, such as asphalt, have a higher thermal capacity than natural surfaces and often exhibit lower albedo, meaning they absorb more sunlight. Combined with heat contributions from car engines, air conditioning systems, and other sources, these materials significantly elevate urban temperatures compared to surrounding suburban and rural areas, thereby contributing to the formation of UHIs (Phelan et al. 2015). Using a zero-order urban thermal model, Phelan et al. (2015) discuss the mechanisms underlying the formation of UHIs. They explain that the primary factor impacting surface temperature is the balance of heat fluxes at the surface, including the contribution of anthropogenic heat. The authors further examine higher-order urban thermal models that account for spatial variations in surface properties and energy exchanges.

Various methods have been employed by scientists to study UHIs, including satellite remote sensing techniques (Phelan et al. 2015). While in-situ observational data is crucial for atmospheric and Earth science studies, they are often limited to specific locations and times. In contrast, modeling approaches provide information on a broader spatial and temporal scale, as demonstrated in the literature (Voogt and Oke 2003). Several modeling approaches have been developed to model the UHI effect. One of the first contributions was by Oke (1982), who proposed a transient energy balance framework to predict urban surface temperatures. More recently, the development of complex mesoscale meteorological models, such as the Weather Research and Forecasting (WRF) model (Skamarock et al. 2019), enabled researchers to explore UHI dynamics in greater detail. These numerical models provide a powerful tool for simulating interactions between urban surfaces, atmospheric dynamics, and heat distribution, offering new insights into the intensity and spatial variations of UHI across different urban environments (Mirzaei 2015; Mirzaei and Haghighat 2010; Weng 2009).

This study adopts the latter approach, using two sets of week-long simulations (explained in detail in the “Methods and approaches” section) by implementing a multiscale configuration of the WRF model to examine the effects of the UHI phenomenon at an exceptionally high resolution across the Hampton Roads (HR) region of VA, USA (Fig. 1). To capture neighborhood-scale heat variations, we utilize the Large-Eddy Simulation (LES) capability within WRF (WRF-LES) to conduct real-case simulations, refining the model resolution to 150 m. However, accurately simulating atmospheric processes remains a challenging task, particularly in complex environments such as our study region, where coastal influences add further complexity to the atmospheric dynamics. Numerous studies have explored the turbulence-resolving capabilities of WRF-LES, primarily through idealized simulations that lack coupling with mesoscale processes or real-world meteorological forcing. Early efforts focused on refining subgrid-scale (SGS) turbulence models to enhance simulation accuracy. For instance, Moeng et al. (2007) introduced a new SGS turbulence scheme to mitigate biases in temperature and vertical velocity within two-way nested LES runs.

Subsequent advancements aimed to further refine the representation of SGS turbulence in WRF-LES. For instance, Mirocha et al. (2010) and Kirkil et al. (2012) incorporated several sophisticated turbulence models, including the nonlinear backscatter and anisotropy model, the Lagrangian scale-dependent dynamic model, and the dynamic reconstruction model (Chow et al. 2005). These enhancements demonstrated notable improvements in idealized LES simulations compared to the traditional Smagorinsky formulation with a constant coefficient. Despite these advancements, WRF-LES simulations in nested real-world simulations remain scarce.

Compared to mesoscale simulations using standard planetary boundary layer (PBL) parameterizations in WRF, the LES configuration offers a more accurate representation of turbulent structures and spatial variability by explicitly resolving large eddies. While mesoscale WRF provides computational efficiency, it relies on parameterized turbulence and often underestimates subgrid variability, especially in complex surface environments. Hybrid RANS-LES approaches, such as Detached Eddy Simulation and its variants, reduce computational expense by blending RANS near surfaces with LES farther downstream, providing a trade-off between detail and cost; however, they often struggle to resolve near-surface turbulence accurately due to interface inconsistencies (Chaouat 2017). In contrast, WRF-LES has been shown in prior studies (Muñoz-Esparza et al. 2014) to better capture boundary-layer dynamics and urban dispersion features, making it well-suited for high-resolution urban meteorological applications despite its higher computational cost.

This study is structured around three key objectives to address the critical challenge of urban climate dynamics in complex geographical regions such as the Hampton Roads region. First, the research explores a range of mesoscale to LES configurations within the WRF model. Considering the inherent challenges of LES modeling in real-case applications, our primary goal is to determine the optimal configuration for this domain, to assess whether meaningful LES modeling of a real-world simulation is feasible, and to explore the technical aspects of this modeling approach in a region characterized by complex terrain that includes coastlines. This process will also address specific challenges associated with using LES within the WRF framework, ensuring that the selected configuration produces the most reliable results for the study area.

Second, this study aims to identify and simulate extreme heat episodes to analyze UHIs within the region at the neighborhood level. A comprehensive analysis of these heat phenomena is conducted, including the identification of their spatial and temporal patterns to better understand their potential impacts on the local environment and population.

Third, we examine the relationship between heat exposure and socio-economic factors at the neighborhood scale within Hampton Roads, with a specific focus on median household income. Median income is selected because it provides a direct and quantifiable link to heat exposure through energy demand and the associated economic burden of cooling. This objective provides valuable insights into how different communities experience urban heat stress and the implications for public health and urban planning.

We have selected the designated study domain (Fig. 1) as the focus of this study due to its high population density and history of socioeconomic challenges and health disparities (Michel et al. 2024). These long-standing inequities have left disadvantaged communities poorly equipped to adapt to environmental stressors, such as heat and air pollution from local sources. For example, Hsu et al. (2021) examined the relationship between UHI intensity and demographic factors in major US cities, finding that people of color and low-income households are disproportionately exposed to a higher UHI intensity. Given its unique environmental and social dynamics, the study domain (urban areas within HR) is ideal for examining the UHI phenomenon. This research represents the first phase of a broader, long-term study aimed at integrating high-resolution modeling results with atmospheric chemistry to explore air pollution patterns within the region. In this paper, we will present our model configuration data in the “Methods and approaches” section, discuss our findings in relation to our research questions, and present the results in the “Results and discussions” section, with a conclusion in the “Conclusions” section.

## Methods and approaches

In this section, we will discuss our modeling approach and explain the WRF-LES model configuration for our designated domain in the “WRF-LES model configuration” section and the statistical metrics used in this study in the “Statistical methods” section. Next, we will discuss the performance of our model configuration for three meteorological variables available from the model and observations in the “Model performance evaluation” section. Next, we will address the datasets and methods we have used for UHI and socioeconomic analysis in our study in the “Socio-economic data and approaches” section.

### WRF-LES model configuration

In this study, we used the WRF model version 4.3 in LES mode (WRF-LES) to create a high-resolution real-case simulation of atmospheric conditions around HR. WRF was developed by the U.S. National Science Foundation National Center for Atmospheric Research (NCAR) and is one of the most widely used NWP models (Skamarock et al. 2019). WRF-LES is an advanced turbulence-resolving capability that allows one to conduct simulations of realistic, evolving boundary layers influenced by large-scale flows (National Center for Atmospheric Research (NCAR) 2025). Given the complex nature of the atmosphere and challenges associated with a real-case simulation of the atmosphere at LES scale, previous studies with LES were performed primarily on idealized cases (Kirkil et al. 2012; Mirocha et al. 2010; Moeng et al. 2007).

Our goal is to create a real-case simulation by conducting a multiscale modeling approach where we set up the WRF model to include two parent mesoscale domains (d01 and d02) with horizontal grid spacings of 4050 m and 1350 m, respectively, downscaled to 150 m grid spacing for the innermost (LES) domain (d03). We selected a time step of 20 s for the parent domain (4050 m resolution), following WRF guidelines recommending a time step between 3 to 6 times the horizontal grid spacing ($\Delta h$) (Skamarock et al. 2019). An adaptive time-stepping scheme was applied, allowing the model to adjust the time step dynamically based on the Courant number, improving computational efficiency while maintaining numerical stability in the high-resolution LES domain. We use a one-way nesting approach to avoid LES interference with the mesoscale solution. In a multiscale approach, the LES domain (nested within d02) receives its boundary conditions from the solutions provided at the lateral boundaries with its parent domain. Meanwhile, we use data from the North American Mesoscale (NAM) Forecasting System with a 12 km grid spacing (National Oceanic and Atmospheric Administration (NOAA) 2006) to provide initial and lateral boundary conditions to the outermost domain (d01) every 6 h. The default NAM dataset provides sea surface temperature (SST) at a 12 km resolution, while water bodies in the study domain are in order of a few kilometers. This means the temperature of the water bodies in the domain is effectively treated the same as the ocean temperature, limiting spatial SST variability. To address this, we incorporate a higher-resolution (0.01-degree,  1 km) global daily-varying SST dataset from NASA Jet Propulsion Laboratory (JPL) (2015), improving differential heating and sea/land breeze representation in the domain. The JPL-SST data, available at 1 km spatial resolution, are dynamically interpolated to the 150 m WRF grid and updated at every model time step to ensure that the lower boundary condition reflects the most recent SST conditions throughout the simulation.

**Table 1:** Details of the WRF-LES modeling system

Simulation days	June 22nd to June 30th, 2024 and July 5th to Jul 11th, 2024
Number of domains	3 domains, including two meso-scale (d01 & d02)
	and one micro-scale domains (d03)
Horizontal grid spacing	d01: 4050 m, d02: 1350 m, and d03: 150 m
Vertical layers	53 layers
Simulation period	14 days total (runs divided into 72 and 51 h segments
	for meso-scale and LES domains, respectively)
Model time step	20 s and 4 s for the parent and LES domains, respectively
Spin-up time	24 and 3 h for the meso-scale and LES domains, respectively
Initial/boundary conditions	NAM reanalysis data, 6-hourly, 12 km grid spacing (National Oceanic and Atmospheric Administration (NOAA) 2006)
Sea Surface Temperature	NASA JPL 1 km resolution SST (NASA Jet Propulsion Laboratory (JPL) 2015)
	updated dynamically using time-varying SST
Land Surface Model	Noah-modified 21-category
	IGBP-MODIS (Niu et al. 2011)
Planetary Boundary Layer Scheme	YSU (Hong et al. 2006)
Shortwave/longwave radiation scheme	RRTMG
WRF-LES grid size	325 $\times$ 343 grid cells

Other model configurations include utilizing the Noah-MP land surface model (LSM) (Niu et al. 2011), with the revised MM5 surface layer scheme (Jiménez et al. 2012). Noah-MP LSM provides a multi-parameterization framework governing land–atmosphere interactions. These processes include vegetation dynamics, soil moisture, snow accumulation and melt, and energy balance, among others. By integrating multiple physics-based processes, Noah-MP enhances the realistic representation of environmental conditions (Hamitouche et al. 2025). In addition, Noah-MP LSM can account for the presence of impervious surfaces (e.g., roads, rooftops) in urban areas, which significantly affect runoff and surface temperatures. In addition, we initiate our model using the urban fraction data in 30 m resolution by the National Land Cover Dataset (NLCD2011, Homer et al. 2015) within the LSM.

Additional physics options include the Dudhia shortwave radiation scheme (Dudhia 1989), the Rapid Radiative Transfer Model (RRTM) for long-wave radiation (Mlawer et al. 1997), and the Thompson et al. microphysics scheme (Thompson et al. 2008). We use the Yonsei University planetary boundary layer (PBL) scheme (Hong et al. 2006) with topographic correction for surface winds (Jiménez and Dudhia 2012) for all domains except the LES domain, where we turn off the PBL scheme and activate the Deardorff three-dimensional SGS turbulence model (Deardorff 1980). The SGS stress model in LES effectively parametrizes the smaller, higher frequency eddies within the inertial subrange of three-dimensional turbulence (Xu et al. 2024). The details of the WRF simulations are represented in Table 1. Our domain of study is illustrated in Fig. 1.

To prevent numerical instability, where small numerical errors can accumulate over time and lead the model away from physically realistic solutions, each mesoscale simulation runs for 72 h, with the LES domain initialized after the first 21 h to reduce the influence of initial conditions on the turbulence-resolving domain. Specifically, we start each mesoscale run at 00:00 UTC and initialize the LES domain at 21:00 UTC on the same day. The simulations then continue for an additional 51 h, from which we discard the first 3 h to allow the LES domain to spin up, leaving 48 h of usable LES output for each run. As a result, the LES simulations span a total duration of 14 days, or 336 h, over two sets of simulations. The first set of LES simulations (excluding the spin-up period) covers June 22–30, 2024, and the second set covers July 5–11, 2024. Based on observations from the Norfolk International Airport (ORF, https://weatherspark.com), these two periods include the hottest heat episodes when the heat persisted for at least five continuous days, which makes these two periods suitable for our UHI study.

Furthermore, we selected areas of interest that are located well within the interior of the LES domain. By keeping our analysis away from the domain boundaries, we minimize the influence of boundary conditions on the results, helping to ensure that the simulated heat episodes and UHI patterns are driven by local atmospheric processes rather than by artificial effects from the model edges.

For the rest of this study, we will focus our analysis on d03 (the LES domain) as our goal is to understand if meaningful real-case LES modeling is feasible for our domain of interest.

**Fig. 2:**
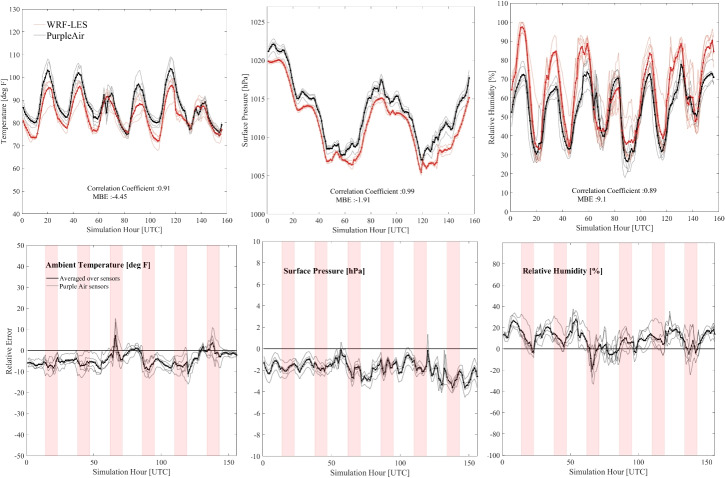
The top row shows time series of WRF-LES model outputs (red lines) compared with measurements from Purple Air sensors (black lines). Both datasets are spatially averaged over the five sensor locations. The bottom row displays the relative error between the WRF-LES and Purple Air data (WRF-LES minus Purple Air). From left to right, the columns represent ambient temperature ( $^\circ F$), barometric pressure (hPa), and relative humidity (%). The WRF-LES outputs are taken at 2 m for temperature, 12 m for pressure, and 2 m for humidity. Gray lines represent the time series at individual sensor locations, while the pink shaded areas in the bottom row mark daytime hours (09:00 to 18:00)

### Statistical methods

To evaluate the performance of the WRF-LES model against observations in the next section, we used widely applied statistical metrics: the * Pearson correlation coefficient (CC)*, *mean bias error (MBE)*, and *root mean square error (RMSE)*. These metrics quantify how well the model captures the temporal variations and magnitudes of key atmospheric variables.

- **Correlation Coefficient:** Measures the strength of the linear relationship between model predictions and observations:
  1 $$\begin{aligned} CC = \frac{\sum _{i=1}^{N}(M_i - \overline{M})(O_i - \overline{O})}{\sqrt{\sum _{i=1}^{N}(M_i - \overline{M})^2 \sum _{i=1}^{N}(O_i - \overline{O})^2}} \end{aligned}$$
- **Mean Bias Error:** Indicates the average tendency of the model to systematically overestimate or underestimate observations:
  2 $$\begin{aligned} MBE = \frac{1}{N} \sum _{i=1}^{N}(M_i - O_i) \end{aligned}$$
- **Root Mean Square Error:** Reflects the overall magnitude of the error:
  3 $$\begin{aligned} RMSE = \sqrt{\frac{1}{N} \sum _{i=1}^{N}(M_i - O_i)^2} \end{aligned}$$

where $M_i$ and $O_i$ represent the modeled and observed values at time step *i*, respectively, $\overline{M}$ and $\overline{O}$ are their respective mean, and *N* is the total number of observations.

These metrics were calculated for temperature, barometric pressure, and relative humidity across five Purple Air sensors Fig. 1 and for wind speed across three MesoWest stations located within the LES domain, as described in the next section and in Section A.

In addition to equations above, and to be able to quantify the relationship between income level and heat exposure, we have used two additional metrics such as the total cooling energy demand (Eq. 4) and percent increase in the total cooling energy demand (Eqs. 5 and 6) which are described in detail the “Socio-economic data and approaches” section.

These metrics are used and discussed in their relative sections throughout this article.

### Model performance evaluation

The WRF model performance has been evaluated several times in the literature (Giannaros et al. 2017; Mohan and Sati 2016) since its initial release in 2000. Nevertheless, before we start our analysis, we perform a brief model evaluation for the most important and available variables from measurements to ensure the quality of our results and present the uncertainties. Since the domain of the study includes only a limited region in HR, there are no measurement stations available from the EPA’s Air Quality System (AQS) monitoring sites within the LES domain. Thus, for the ground measurements, we rely on the measurements from five Purple Air sensors that we detected within our domain, which are publicly accessible (https://www.purpleair.com). The locations of the Purple Air sensors are shown in Fig. 1 with dark circles.

**Fig. 3:**
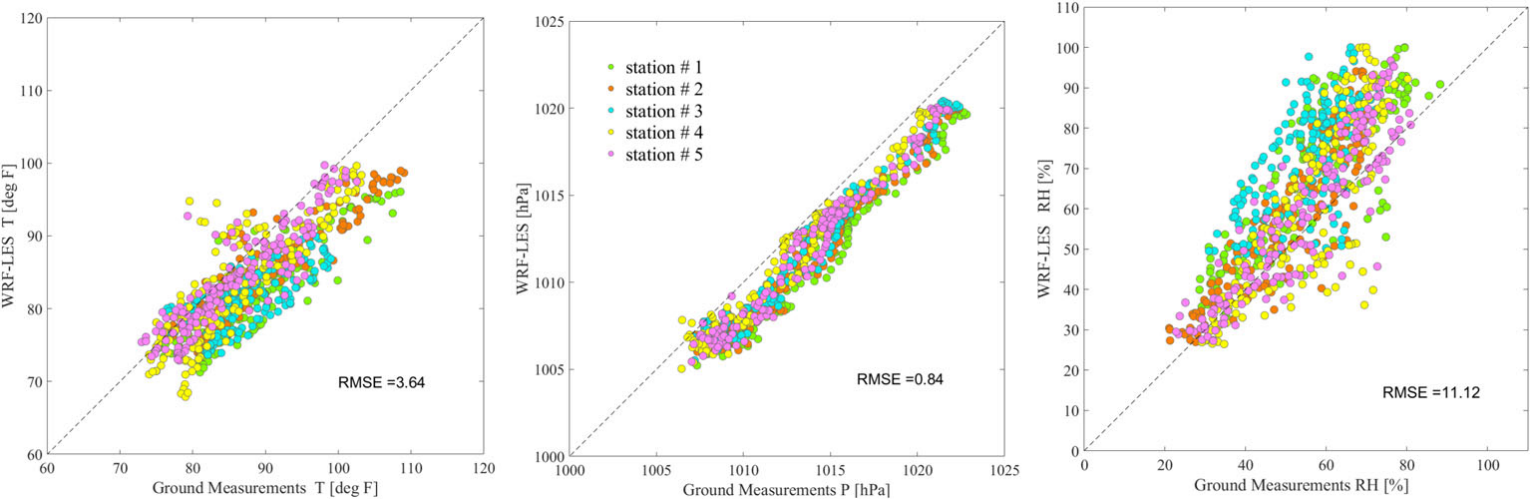
WRF-LES model output (y-axis) compared with the collected data from the Purple Air sensors (*x*-axis) at five locations indicated with distinct colors in the scatter plots for ambient temperature [ $^\circ F$] (left), barometric pressure [hPa] (middle), and relative humidity [%] (right). The WRF-LES outputs are extracted at 2 m, 12 m, and 2 m heights above ground for temperature, pressure, and humidity, respectively

Similar to the WRF-LES model outputs, the Purple Air data are provided hourly. A time-series comparison between the WRF-LES outputs and the data collected from five Purple Air sensors is illustrated in Fig. 2 (top row), for three related variables, i.e., ambient temperature, barometric pressure, and relative humidity. In addition, the relative error of the model (*d*) at every simulation hour is illustrated in Fig. 2 (bottom row). These figures are useful to determine the simulation accuracy as a function of the time of day.

To evaluate the performance of the model, we study three metrics, i.e., *CC* (Eq. 1), MBE (Eq. 2), and RMSE (Eq. 3), as described in the “Statistical methods” section. As illustrated in Fig. 2, it appears that WRF-LES performs satisfactorily for all three variables with *CC* and mean errors within the expected range as described in the rest of this section.

For ambient temperature, WRF-LES shows a strong performance in capturing the trends of the temperature, with a correlation coefficient of 0.91, with a tendency to under-estimate the temperature. The MBE is $-$4.45 $^\circ F$, which indicates a systematic underestimation of around 5% across the monitoring stations within the designated domain, suggesting that the extreme heat and consequently the energy burden studied in this article will be based on an under-estimated temperature profile from the model, and therefore a conservative assumption. This suggests even more extreme conditions in the real world compared to the simulated results, which ensures that our results are not likely to exaggerate the high-heat conditions, but potentially underestimate them.

For the barometric pressure at the surface, the WRF-LES model systematically underestimates pressure ($MBE = - 1.91$ hPa). Despite the slight underestimation, there is a robust correlation of 0.99 between the WRF-LES outputs and ground pressure measurements, indicating the strong performance of the model in predicting atmospheric pressure.

Although the WRF-LES model underestimates both pressure and surface temperature, it slightly overestimates relative humidity ($CC \approx 89\%$ and $MBE \approx 9\%$). This overestimation in humidity is partly due to the underestimated temperature, indicating that the vapor mixing ratio is closely estimated by the model. Assuming the Purple Air sensors are well calibrated for humidity measurements, the model’s relative humidity at 2 m height exceeds observational values. Although this discrepancy could influence the calculations of the thermal heat index, Fig. 2 (bottom row rightmost figure) shows that the positive bias for RH follows a pattern where the largest errors are associated with the night hours compared to the day hours (shown with vertical pink patches), when the hottest temperatures occur. Given this distinct pattern and the fact that the WRF-LES model underestimates temperature, the overall impact on heat index estimates is likely mitigated. Furthermore, studies suggest that while relative humidity errors can affect heat index calculations, temperature biases often play a more dominant role in perceived heat stress (Anderson and Bell 2013; Buzan et al. 2015). An additional wind speed analysis was conducted using data from three MesoWest stations (https://mesowest.utah.edu/). Based on Fig. S1 and the accompanying statistics, the model shows strong agreement with observed wind speed measurements ($MAE=1.05 m/s, RMSE=1.33 m/s, and CC=79\%$).

While Fig. 2 serves as an indicator of model performance, some spatial features may be lost due to averaging across five sensors. To address this, Fig. 3 presents the relationship between all data points co-located in space and time between the model and observations. Each panel in Fig. 3 displays the *RMSE* value for that variable, providing another key metric for evaluating the model performance. Figure 3 indicates a consistent pattern with results in Fig. 2 across all data in five stations, confirming the presented model performance across all the available sensors.

The figures and statistical results presented here validate the WRF-LES model’s effectiveness as well as uncertainties in simulating the complex and dynamic conditions of a real scenario within the study domain over the analyzed period. Given an acceptable performance of the model, we proceed with this configuration for further analysis. In the “Meso-scale to LES configurations” section, we provide a detailed discussion of this setup and explicitly outline the unreliable configurations that were identified and excluded to ensure the integrity of our results.

### Socio-economic data and approaches

To explore the relationship between UHI and socioeconomic factors, we focused on three key variables: the percentage of the African American population, median household income, and median housing values. Data analysis was performed using ArcGIS Pro 3.X (Environmental Systems Research Institute, Inc 2024). To integrate the modeled temperature data with socioeconomic data, we first converted the temperature data into point layers. We then applied the Inverse Distance Weighting (IDW) method to interpolate these points into 30-by-30-meter grid-based raster layers, providing a more precise match to the smaller census tracts in higher population density areas. Given HR’s location, portions of the study area are covered by water. To ensure the analysis focused on land areas, the water regions were masked out from the interpolated temperature layers.

Socioeconomic data were sourced from the U.S. Census Bureau (U.S. Census Bureau 2024), including race (B02001), median household income over the past 12 months (B19013), and median value of owner-occupied housing units (B25077) from the American Community Survey at the census tract level. The race data were used to calculate the percentage of the African American population by dividing the number of individuals identified as African American alone by the total population. To examine the relationship between temperature and socioeconomic factors were aggregated to the census tract level using the Zonal Statistics tool, which computed the mean and median temperature values within each tract. These values were then extracted and merged with the socioeconomic data using census tract geographic identifiers (GEOIDs). Only the census tracts entirely within the spatial range of the temperature data were included in the analysis, totaling 320 tracts.

While we explored the spatial correlation between temperature and all three socioeconomic variables, our analysis specifically focuses on median household income. Income offers a direct and quantifiable link to heat exposure through energy demand and the economic burden of cooling. For this reason, we have discarded the other two variables and will continue our analysis focusing solely on income level.

To establish a connection between a physical variable (ambient temperature/heat index) and economic factors (e.g., median income level), we define and utilize cooling energy demand as a key variable, as it directly influences both sensible temperature and economic conditions by determining the energy required for residential cooling. The cooling demand is estimated under the assumption that air conditioning is necessary when the heat index exceeds 75 $^\circ F$, based on human comfort thresholds. Specifically, we focus on total cooling degree hours (CDH), defined as the integrated temperature differences between the simulated heat index and the 75 $^\circ F$ threshold across the simulation period, calculated at every grid cells, as presented in Eq. 4.

4 $$\begin{aligned} CDH_m = \int _i^n (\tau _i - T_b) dt \end{aligned}$$

where *CDH* is the total cooling demand (cooling degree hours) in gridcell *m*; *i* is the time index, $\tau$ is the heat index, and $T_b$ is the base temperature (75 $^\circ F$). We computed the CDH using 30-min interval outputs from WRF, with CDH representing the time integral of these calculations. Accordingly, in Eq. 4, $\Delta t$ is set to 0.5 h to match the model output interval.

We do not convert this variable into a direct financial state as we would need to make extra assumptions for the electricity price and the hours it would take for the cooling equipment to cool a certain location. The cooling demand calculated here is a variable relative to the energy required to cool down a space in different parts of the domain.

In addition, we have quantified the percent increase in cooling energy demand across the domain relative to the minimum land-based requirement by normalizing the values with respect to the minimum inland cooling demand (excluding water-covered areas) using the following equations:

5 $$\begin{aligned} CDH_{n_{ij}}= &  {CDH_{ij}\over CDH_{min}} \end{aligned}$$

6 $$\begin{aligned} CDH_{np_{ij}}= &  (CDH_{n_{ij}} - 1) \times 100 \end{aligned}$$

where $CDH_{ij}$ represents the cooling degree hours at grid cell ij, $CDH_{n_{ij}}$ is the normalized CDH at grid cell ij, $CDH_{np_{ij}}$ denotes the normalized CDH expressed as a percentage, and $CDH_{min}$ is the minimum inland CDH value.

## Results and discussions

### Meso-scale to LES configurations

The multi-scale modeling of a diurnal cycle under real-world conditions in the WRF model, using the cell-perturbation method, was first presented by Muñoz-Esparza et al. (2014). Transitioning from the mesoscale to the microscale in a real-world multiscale modeling scenario can be challenging, particularly because the LES domain must develop turbulence on its own, as the atmospheric inflow from the parent domain with a PBL scheme is non-turbulent. Additionally, the shift from one-dimensional turbulent diffusion in the mesoscale model to three-dimensional LES mixing does not always lead to an immediate development of turbulence in the LES domain. The cell-perturbation method applies stochastic perturbations in the potential temperature field within a region near the inflow boundaries of the LES domain. These finite-amplitude perturbations result in an accelerated transition to fully developed turbulence within the LES domain (Lee et al. 2019; Muñoz-Esparza et al. 2014).

**Fig. 4:**
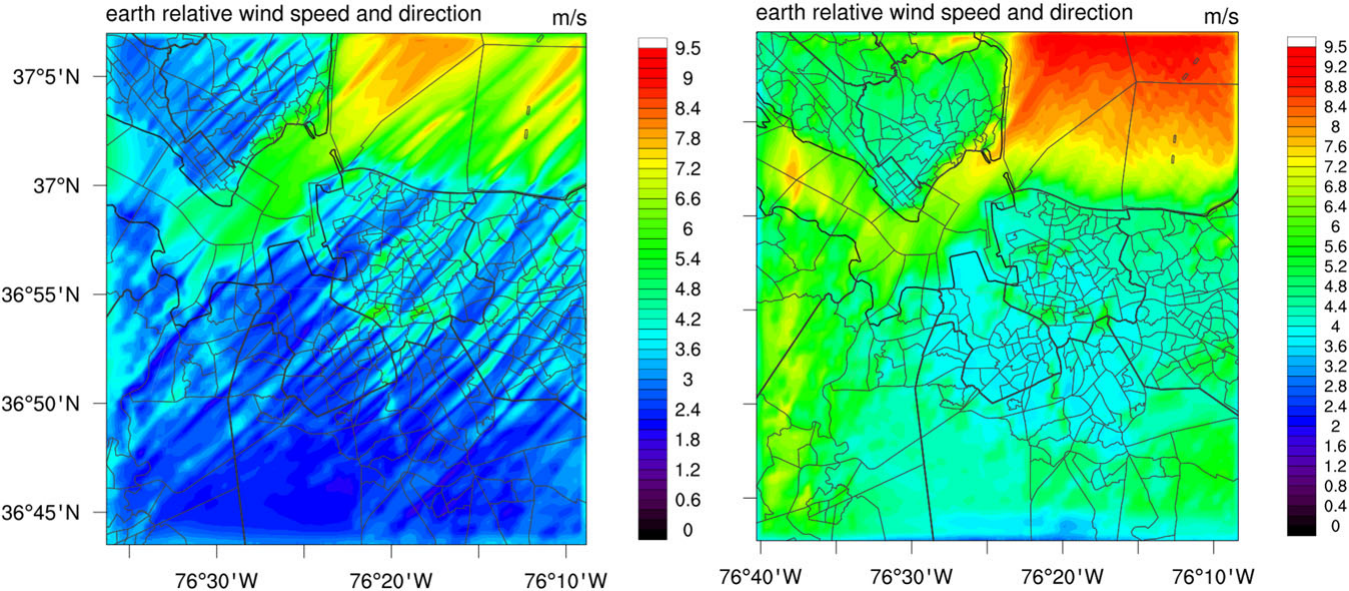
Wind speed at hour 12 of the control simulation (left) without the cell-perturbation method, and simulation with the cell-perturbation method (right). The low-high wind speed patterns that resemble a wave pattern in the control simulation (left) are eliminated in the final simulations using the cell-perturbation method (right)

In our initial simulations (control case), conducted without implementing the cell-perturbation method, we identified notable artifacts in the hourly wind speed outputs. These artifacts appeared as alternating high and low wind speed regimes aligned in parallel, indicative of model instability (e.g., Fig. 4a). Such anomalies (Ching et al. 2014), resembling wave peaks and troughs, were most pronounced during periods of strong convection or transitions between day and night. These patterns were non-physical and likely attributable to inherent model limitations.

Similar artifacts were previously detected in an earlier project, where the activation of wind turbine parameterization within a mesoscale setup introduced noise, ultimately resulting in comparable model artifacts. This issue was discussed in previous work (Golbazi et al. 2022; Golbazi and Archer 2024), highlighting the potential of such artifacts to lead to misinterpretation of physical phenomena if not properly addressed. In addition to the artifacts, our simulations revealed remarkably low wind speeds within the LES domain in our control case, which was an indicator of misrepresentation of the characteristics of the convective boundary layer turbulence. The exact nature of the problem is unclear, but further investigation is beyond the scope of the current study.

To address these discrepancies, we conducted a series of test simulations employing various strategies recommended by model developers, model users, and recommendations documented in the literature. These strategies included adjusting the sixth-order diffusion parameter, reducing the time step, modifying domain resolution, altering the ratio between the parent and nested domains, implementing adaptive time-stepping, and applying analysis nudging for model stability. Despite these efforts, none of the adjustments succeeded in generating more realistic simulations.

In meteorology, numerical modeling falls into two main categories, i.e., mesoscale modeling for large domains and LES for smaller domains. The key distinction is the ratio of the energy and flux containing turbulence scale (*l*) to the spatial scale of the designated domain ($\Delta$). In mesoscale modeling, this ratio ($l/\Delta$) is small and the turbulence is not resolved, whereas in LES, $l/\Delta$ must be large enough to capture turbulence. Advances in computational power now allow for very fine-mesh mesoscale modeling, but neither traditional LES nor mesoscale approaches are designed for the intermediate range—“terra incognita”—where *l* and $\Delta$ are of the same order (Wyngaard 2004). Consequently, even with nested LES in WRF, resolving small-scale turbulence remains challenging. To avoid the influence of the challenges within “terra incognita” on the LES domain, we increased the ratio between the parent and nested domain. Although this effort helped decrease the model instability and decrease the model artifacts, it did not sufficiently address the problem.

Ultimately, we adopted the cell-perturbation method introduced by Muñoz-Esparza et al. (2014) in addition to the improvement from skipping the gray zone. All other settings remained the same as in the control case. As a result, the artifacts were effectively eliminated, as evidenced by the improved hourly outputs presented in Fig. 4b. We find that the implementation of the cell-perturbation method results in more realistic wind speed outputs (Fig. 4b), a good model performance (as discussed in the “Model performance evaluation” section), and therefore simulations that more accurately represented realistic conditions in the LES domain, thus meeting one of our primary objectives to assess the feasibility of high-resolution modeling with reliable accuracy in this context.

### Urban heat islands in Hampton Roads

After determining the optimal model configuration for the Hampton Roads domain, we proceed to explore the presence of UHIs within the study area. Considering that Norfolk, Hampton, and Newport News in Virginia are relatively small urban areas within the domain, our objective is to determine whether UHI effects are present in this region and to evaluate their intensity and impact on residents. This analysis is particularly important as several communities residing in the area, some of which face significant challenges in accessing resources to mitigate the impacts of extreme heat events. We selected four key parameters to study extreme heat: (1) the 2 m temperature from the model (referred to surface temperature, hereafter), which directly represents extreme heat; (2) the thermal heat index, which accounts for the impact of humidity on human perception; (3) percent time residents experience “Extreme Caution” for heat; and (4) total cooling demand, which reflects the energy required for cooling due to extreme heat.

**Fig. 5:**
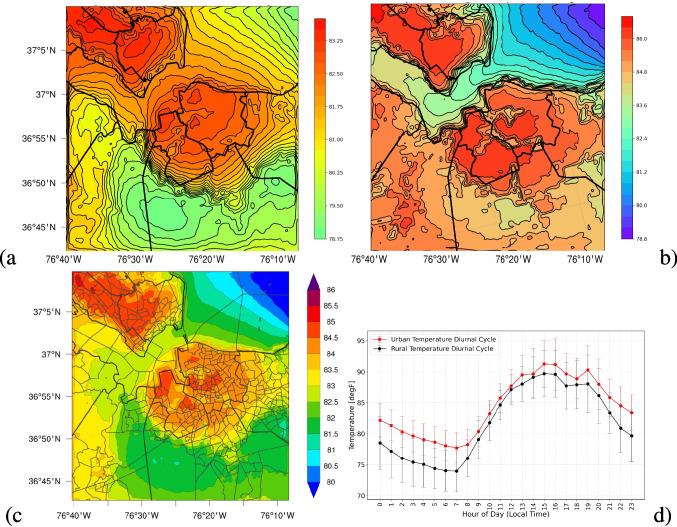
Time-averaged surface temperature [$^\circ F$] for **a** over nighttime hours (18:00–09:00), **b** over daytime hours (09:00–18:00), and **c** over all hours in simulation period. A sample mean diurnal cycle of a random urban vs rural area is shown in **d** with red and black lines, respectively. Contour lines indicate the boundaries of each temperature interval represented by the color bar. Thick black lines mark the boundaries of individual counties, and the medium black lines inland indicate individual US Census tracts

Figure 5c illustrates the time-averaged surface temperature over a 14-day simulation period, while Fig. 5a and b show nighttime (18:00–09:00) and daytime (09:00–18:00) averages, respectively. A comparison between the temperature distributions in this figure with the domain structure in Fig. 1 reveals significantly higher temperatures (up to 6 $^\circ F$ on average) in urban areas such as Norfolk, Hampton, and Newport News compared to rural regions, with the peak temperatures observed in Newport News, VA. To further illustrate this contrast, we analyze the time series of two randomly selected urban and rural locations, as shown in Fig. 5d. In this figure, each data point represents the average temperature at a given hour of the day, over the simulation period. The results indicate a temperature difference of up to 5 $^\circ F$ between the urban and rural areas. This disparity is most pronounced at night, when rural areas cool down while urban areas retain heat, demonstrating the persistence of the UHI effect (Fig. 5a and d). This difference is likely higher in reality, as we do not account for all added anthropogenic heat.

While ambient temperature provides a direct measure of thermal conditions within a given domain, the heat index (HI), also referred to as the apparent temperature, quantifies the perceived temperature by accounting for both air temperature and relative humidity. Recent studies highlight the necessity of accounting for humidity in UHI assessments, as it greatly influences human-perceived temperature, often differing from the measured ambient temperature (Tuholske et al. 2021; Heo and Bell 2019). Humidity affects thermal comfort by altering the body’s ability to regulate heat through perspiration, making its inclusion essential in evaluating heat stress.

To provide a more comprehensive assessment of thermal comfort, we analyze the heat index as defined by NOAA (National Oceanic amd Atmospheric Administration, National Weather Services 2025). The HI is calculated as a function of both ambient temperature and relative humidity, with a well-established positive correlation; as either variable increases (or decreases), the heat index correspondingly increases (or decreases). The precise formulation of the heat index follows the methodology outlined in National Oceanic amd Atmospheric Administration, National Weather Services; Weather Prediction Center (2025). Furthermore, NOAA classifies HI values into different risk categories for public health, ranging from “Caution” to “Extreme Danger.”

**Fig. 6:**
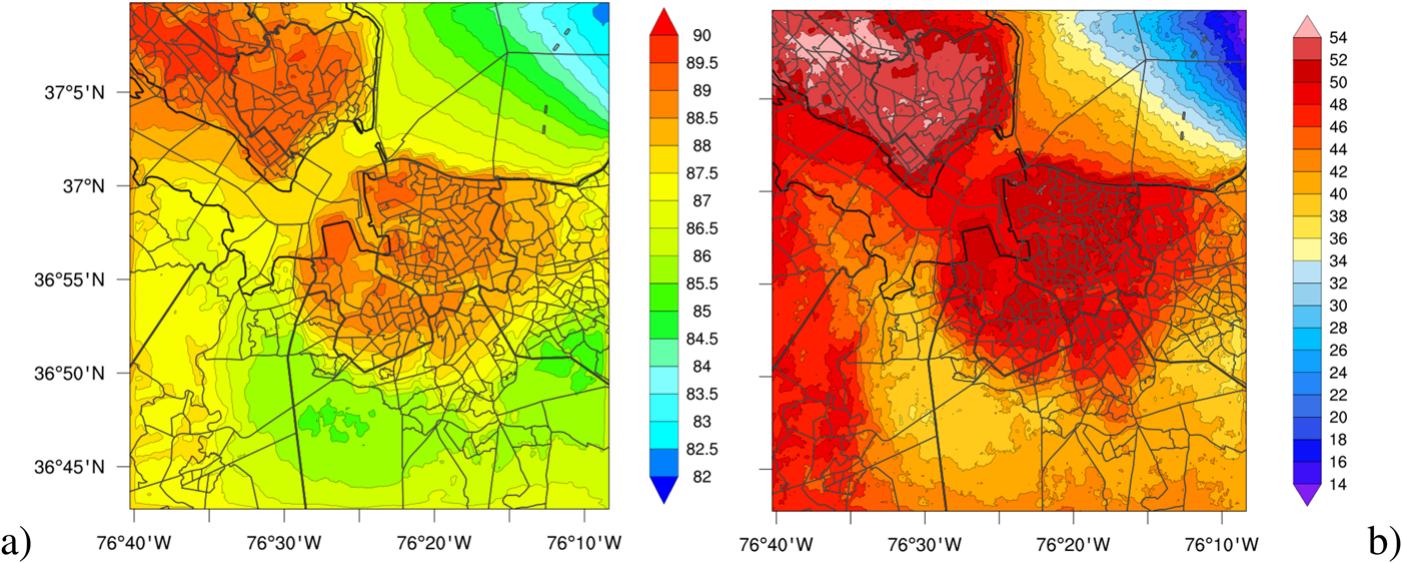
Time-averaged thermal heat index across the domain (**a**), and the percent time when thermal heat index exceeded 90 $^\circ F$, defined as “Extreme Caution” by NOAA (National Oceanic amd Atmospheric Administration, National Weather Services; Weather Prediction Center 2025) in **b**. Contour lines indicate the boundaries of each temperature interval represented by the color bar. Thick black lines mark the boundaries of individual counties, and the medium black lines inland indicate individual U.S. Census tracts

To study the impacts of heat risk on residents, we compute the HI at each grid cell within our domain and present the spatial distribution of the time-averaged HI in Fig. 6a. The HI patterns in this figure align well with our findings from Fig. 5c. To evaluate the frequency of exposure to hazardous heat levels, we generate a spatial map indicating the percentage of time that populations within the study domain experience HI values exceeding 90 $^\circ F$, corresponding to the thresholds of “Extreme Caution,” “Danger,” or “Extreme Danger.” This analysis, shown in Fig. 6b, reveals that in rural areas, HI exceeds the “Extreme Caution” threshold $35-42\%$ of the simulated time. In contrast, urban residents experience HI above this threshold at least $50\%$ of the time, highlighting exacerbated heat in densely populated urban areas and energy sources.

### Socioeconomic impacts of the heat islands in HR

The UHI effect amplifies trends driven by climate change toward longer, stronger, and more frequent heat extremes. Due to a history of race-based urban planning policies, certain communities experience greater environmental inequality, making them disproportionately susceptible to extreme urban heat and its worsening impacts under climate change.

Given that this study focuses on high-heat episodes and UHIs, it is crucial to consider their impact on energy consumption across different neighborhoods. Increased temperatures drive higher cooling demands, disproportionately affecting lower-income communities that may struggle with energy affordability. Research has shown that low-income households face physical energy insecurity and often allocate a larger share of their income to energy costs, increasing the financial burden during extreme heat events (Drehobl and Ross 2016; Hernández 2016). Physical energy insecurity refers to inadequacies in a home’s physical infrastructure that compromise thermal comfort, expose residents to harmful conditions, and drive up energy costs (Hernández 2016; Shapira and Teschner 2023).

To explore these disparities, we analyze socioecomic factors within the study domain, examining their correlation with UHIs and energy burden. By understanding these relationships, our aim is to highlight the socioeconomic dimensions of UHIs and identify the population in the domain that may face the greater challenges in mitigating extreme heat.

**Fig. 7:**
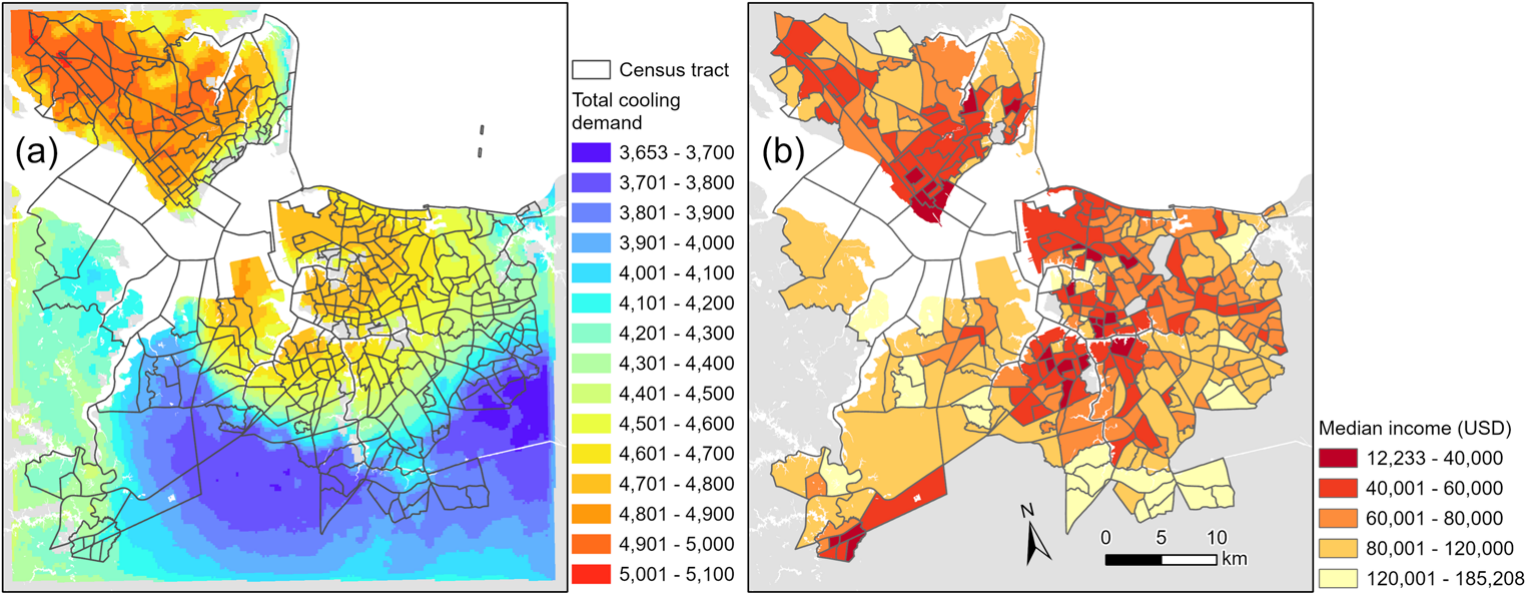
The left panel illustrates the total cooling demand (CDH) as calculated with Eq. 4 in degree hours, defined as the integrated cooling degrees to base temperature (75 $^\circ F$) over the simulation period (**a**). The right panel shows a map illustrating median income of study area at census tract level (**b**)

We calculated the spatial correlation between three socioeconomic factors, i.e., percentage of African American population, median household income over the past 12 months, and median value of owner-occupied housing units driven from the U.S. Census Bureau, and the heat index in domain. The interpolation of these parameters is described in the “Socio-economic data and approaches” section. We found that the strongest spatial correlation (a negative correlation) exists between median household income and the thermal heat index (not shown) in this domain. The negative correlation between these variables suggests that lower-income households experience greater exposure to extreme thermal conditions. This pattern aligns with findings in the literature (Harlan et al. 2006; Mitchell and Chakraborty 2014) and indicates that lower-income households tend to reside in high-density urban areas characterized by the UHI effect, whereas higher-income families are more likely to live in less thermally stressed suburban or rural regions. While this relationship underscores the burden on economically disadvantaged communities to mitigate heat exposure, concluding a direct causal relationship is not appropriate as socioeconomic factors interact with a range of environmental, infrastructural, and behavioral variables that contribute to thermal vulnerability (Madrigano et al. 2015; Reid et al. 2009).

To this end, we have calculated the total cooling energy demand (CDH) in cooling degree hours (Eq. 4; as described in the “Socio-economic data and approaches” section) to establish a relationship between physical and social parameters through a mutual variable. Figure 7a indicates the total cooling energy required over the designated domain, while Fig. 7b shows the median household income over the past 12 months in the U.S. Census tract level. A simple visual inspection shows that urban areas within the domain require substantially more energy for cooling compared to rural areas.

In addition to visual inspection and based on a normalized map of the energy demand in the LES domain (Fig. S2) that was calculated based on Eqs. 5 and 6, we find that urban areas require up to $\approx 140\%$ more energy for cooling purposes. This contrast is particularly pronounced in the Northwestern portion of the domain, corresponding to the city of Newport News. In addition, this disparity leads to more than double the cooling energy demand for certain urban populations, leading to a greater economic burden, as electricity costs often increase non-linearly with demand due to tiered pricing structures and peak load surcharges (Mosquera-López et al. 2024, 2017).

This is all while the highest fraction of the lower-income families are concentrated within the urban areas with highest energy demand due to the UHI. Consequently, urban residents face significantly higher cooling expenses to mitigate heat exposure, particularly in regions where extreme temperatures persist. Moreover, these estimates assume the presence of adequate cooling infrastructure; in areas lacking access to air conditioning or efficient cooling systems, the compounded effects of heat stress and financial constraints may intensify public health risks.

#### Shortcomings and future direction

We recognize that this is a complex and sensitive topic involving human experiences, and we strongly encourage future research to incorporate the broader impacts of heat risk, as defined in National Weather Service (2025). A comprehensive assessment of heat exposure requires the expertise of epidemiologists, sociologists, and the integration of health data to fully capture its consequences and avoid oversimplification.

We acknowledge the study’s limitations regarding the absence of health and epidemiological data. However, our emphasis here is on refining modeling capabilities as a foundational step. While our model can help reveal spatial disparities in heat exposure, interpreting these patterns in the context of social vulnerability requires careful distinction between correlation and causation. This work serves as a basis for future studies that can build on the presented modeling framework, integrating atmospheric chemistry, health outcomes, and social determinants, alongside subject matter expertise, to enable a more comprehensive and causally-informed understanding of heat-related impacts.

## Conclusions

In this study, we conducted high-resolution numerical simulations of Hampton Roads, Virginia, using the LES configuration of the WRF model at a 150 m resolution through a multi-scale nesting approach, coupling mesoscale and LES domains. Our objectives were to evaluate the feasibility of high-resolution real-case modeling at the neighborhood scale and to investigate the impacts of extreme heat events in the region and their relationship with social-economic factors.

To achieve physically realistic simulations at this fine scale, we addressed key modeling challenges, including numerical noise and inflow from the mesoscale domain at the boundaries. The latter produced erroneous LES wind fields likely due to incorrect scales of turbulent motion, further complicating the simulation process. through extensive testing, we determined that the cell-perturbation method effectively mitigated model artifacts by generating inflow turbulence and ultimately producing more realistic LES results. Model performance was evaluated using observations across the study area.

After establishing a realistic simulation, we examined extreme heat episodes in June and July 2024, focusing on periods with sustained heat for at least five consecutive days. Urban heat islands were identified based on three key metrics: (i) 2-meter ambient temperature, (ii) the NOAA-defined heat index, and (iii) he percent of time individuals experienced the “Extreme Caution” category for heat stress. We observed the largest urban–rural temperature differences at night, when rural areas cooled more rapidly than urban zones.

A spatial correlation analysis revealed that median household income was most strongly (and negatively) correlated with heat exposure, indicating that lower-income communities face greater thermal risk, primarily due to their location in densely populated urban areas.

To further quantify this disparity, we introduced *cooling demand* as a key metric to link ambient temperature with economic factors, calculated as the cumulative heat index exceedance above 75 $^\circ F$, to represent the potential energy burden for cooling. Results showed that urban areas required up to 140% more cooling energy than rural areas, resulting in more than double the potential demand for urban populations. This disproportionate burden is amplified by non-linear electricity pricing and peak demand surcharges. While this metric highlights an environmental concern, additional research is needed to establish causal relationships and assess the financial burden of cooling costs on economically disadvantaged communities.

Future work will build upon these findings by extending the study to neighborhood-scale air quality modeling for the HR region. While technically challenging, this effort is crucial for developing a more comprehensive understanding of the environmental stressors affecting local communities and informing targeted mitigation strategies.

## Supplementary Information

Below is the link to the electronic supplementary material.

Supplementary file 1 (pdf 1243 KB)

## Data Availability

This article has no associated data. The WRF-LES model outputs will be provided upon request.
