# Supplementary material for: High-resolution modeling of extreme heat events with socioeconomic consideration: a real-case WRF–LES approach
Source: Environ Sci Pollut Res Int. 2025 Sep 12;32(36):21666–80. doi: 10.1007/s11356-025-36928-w (PMC12484280; doi:10.1007/s11356-025-36928-w)
Supplement: Supplementary file 1 — (pdf 1243 KB) [file 11356_2025_36928_MOESM1_ESM.pdf]

Appendix

SUPPLEMENTARY INFORMATION

This appendix contains additional material and figures to support the study.

A Model Performance analysis (wind speed)

In addition to the three variables used for model performance analysis using six available stations from purple air stations, we conducted a comprehensive evaluation of model performance for wind speed over the entire two-week WRF-LES simulation period using data from three available stations from the MesoWest stations (https://mesowest.utah.edu/). Figure S1 presents the average wind speed time series, aggregated across three MesoWest stations located within the LES domain, alongside corresponding statistical metrics.

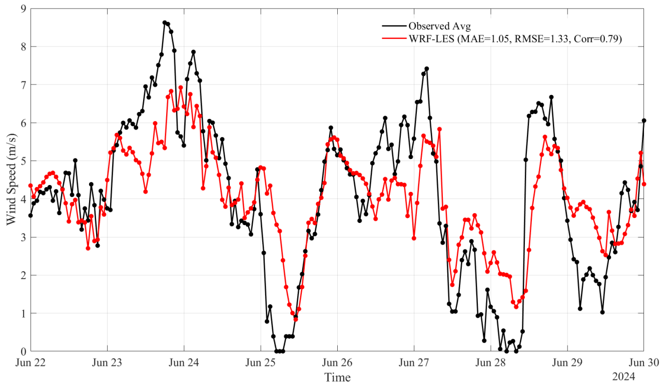

Fig. S1: Figure: Average wind speed time series across all MesoWest stations within the LES domain. This plot compares the hourly-averaged observed wind speeds (black) with WRF-LES modeled wind speeds (red), both aggregated across all available stations. The statistical metrics - MAE, RMSE, and CC - are calculated using these averaged time series to evaluate model performance in capturing temporal wind speed variability.

The results indicate strong agreement between the modeled and observed wind speeds, with the averaged time series demonstrating consistent temporal correlation. Quantitatively, the model has a Mean Absolute Error (MAE) of 1.05 m/s, a RMSE of 1.33 m/s, and CC of 0.79. These statistics confirm the model's capability to reproduce the observed hourly variability in wind speed at the domain scale.

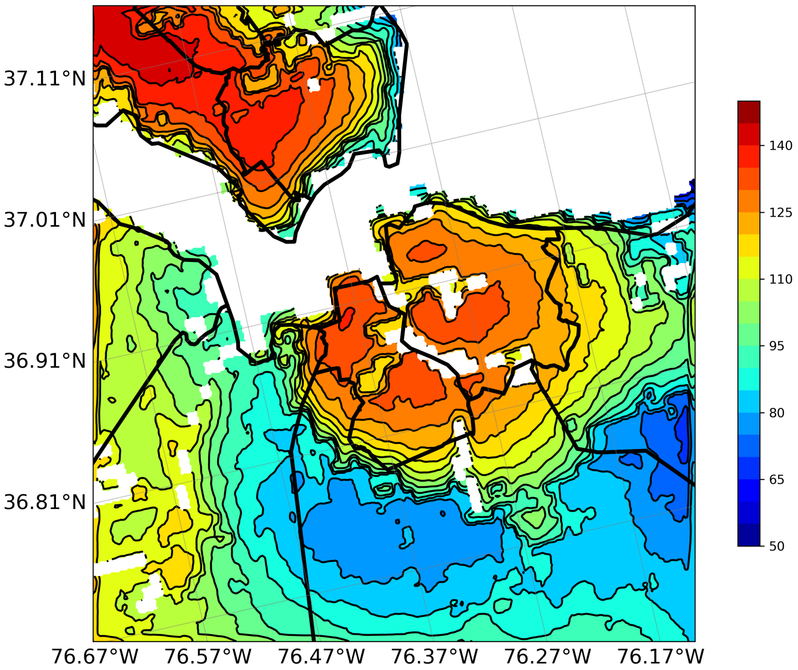

Fig. S2: Cooling energy demand relative to the minimum cooling energy demand over land, expressed as a percentage (%). To avoid distortion from land–water temperature differences, only land areas were included in the calculation.
